# Prevalence and high risk behaviours associated with HCV testing among people who inject drugs: a systematic review and Meta-analysis

**DOI:** 10.1186/s13011-020-00306-1

**Published:** 2020-08-24

**Authors:** Salah Eddin Karimi, Azadeh Bayani, Peter Higgs, Amir-Hossein Bayat, Morteza Hemmat, Elahe Ahounbar, Bahram Armoon, Yadolah Fakhri

**Affiliations:** 1 Social Determinants of Health Research Center, Health Management and Safety Promotion Research Institute, Tabriz University of Medical Sciences, Tabriz, Iran; 2 Student Research Committee, School of Allied Medical Sciences, Shahid Beheshti University of Medical Sciences, Tehran, Iran; 3 Department of Public Health, La Trobe University, Melbourne, Australia; 4 Social Determinants of Health Research Center, Saveh University of Medical Sciences, Saveh, Iran; 5 Substance Abuse and Dependence Research Center, the University of Social Welfare and Rehabilitation Sciences, Tehran, Iran; 6 Department of Environmental Health Engineering, Food Health Research Center, Hormozgan University of Medical Sciences, Bandar Abbas, Iran

**Keywords:** Social determinants, Past treatment attempt, Previous testing, Unprotected sex

## Abstract

**Background:**

Hepatitis C diagnosis could be a gateway to behavioral change and subsequent decline in transmission among people who inject drugs (PWIDs). We assessed the association between the social determinants of PWID, their risk behaviors and hepatitis C testing.

**Methods:**

We searched for studies in English published before May 1, 2020, on PubMed, Scopus, Cochrane, and Web of Science to identify primary studies on the factors associated with hepatitis C virus (HCV) testing among PWID. After reviewing for study duplicates, the full-text of selected articles were assessed for eligibility using Population, Intervention, Comparator, Outcomes (PICO) criteria. i) population: individuals who report injecting drugs; ii) intervention: HCV testing in the past year; iii) comparator: PWIDs who did not have an HCV test; iv) outcome: HCV testing among PWIDs and v) study type: cross-sectional, cohort, and case-control studies. Two independent reviewers (author BA and AB) chose the references in a two-phased monitoring process. The authors gathered data from selected papers, including the surname of the first author, publication date, participant demographic data (age, sex, and level of education) and other characteristics like previous HCV testing, past treatment attempts, duration of injecting drug use and condomless sex. We used fixed and random-effects meta-analysis models to estimate the pooled prevalence, pooled odds ratio (OR), and 95% confidence intervals. The data were analyzed using Stata 12.0 software.

**Results:**

After a detailed assessment of over 12,000 articles, a total of 16 studies containing 38,952 participants met the eligibility criteria. Our findings showed a pooled prevalence rate of 61.01% (95% CI, 34.65–84.32%) for recent HCV testing among PWIDs. Being female (OR = 1.69, 95%CI = 1.13, 2.26), aged > 30 years, (OR = 2.61, 95%CI = 1.66–3.56) having past treatment attempt (OR = 2.24, 95%CI = 1.80–2.68), and reporting a previous test (OR = 2.03, 95%CI = 1.23–2.82). were significantly associated with having a recent HCV test.,,. Finding of present study was that unprotected sex had a negative association with HCV testing. Those PWIDs who had unprotected sex were 0.56 times less likely to have completed HCV testing during last year (OR = 0.56, 95%CI = 0.33–0.78).

**Conclusion:**

Prevention programs that address age > 30 years, being female, past treatment attempt, previous testing of safe sexual practices, are strongly recommended to prioritize HCV risk reduction strategies.

## Background

Hepatitis C virus (HCV) infection is a major public health problem, causing a preventable liver-related morbidity and mortality globally [1–3]. The World Health Organization (WHO) has defined targets for HCV treatment and diagnosis, representing a step towards the goal of elimination by 2030 [4]. Nevertheless, rates of HCV testing, treatment, and linkage to care are still low in many places around the world [5]. A number of social factors impact on treatment access including unstable housing, social stigma, health care providers’ attitudes, criminalization of drug use, and gender [6–9]. Historically, HCV infection has been described as a silent epidemic, with estimations suggesting that half of those infected are not currently aware of their HCV status [10]. People who inject drugs (PWIDs) carry a higher HCV burden than other people, with HCV prevalence in this group being estimated at over 50% [11, 12]. The criminalization of drug use and the political response means custodial settings have high rates of HCV prevalence [13–15].

The sharing of previously used injecting equipment means that PWID are the population most at risk for HCV infection in nearly all middle and high-income countries. Undiagnosed and untreated HCV infection can cause cirrhosis, hepatic decompensation, liver cancer, and death [16]. HCV diagnosis itself could be a helpful strategy [17] in behavioral change [18] and producing a decline of HCV transmission [19, 20].

As part of the elimination strategy, WHO suggests regular testing of PWIDs (at least yearly) [21]. However, such regular HCV testing by active drug users relies on many factors, for example, heightened levels of risk awareness among PWID, availability of testing sites and drug- treatment workers who integrate testing as a normal part of their work [22]. Low rates of HCV treatment uptake among PWIDs have been attributed to barriers at the provider, patient and systems levels. Not least of which have previously been treatment complexity together with limited capacity, the side effects from interferon-based treatment, social stigma and discrimination which may influence the willingness of physicians to provide treatment for PWIDs [23–25].

In addition to financial barriers, other barriers that might prevent PWIDs from accessing HCV treatment and testing include the high levels of discrimination and stigma that may exist inside of conventional healthcare settings, which may influence on patient-provider relationships and the willingness on the part of physicians to treat PWIDs [22, 25].

Despite evidence that the availability of direct acting antivirals has made HCV testing and treatment more accessible [26] PWIDs continue miss out. Making HCV (anti-HCV) and verifying chronic HCV infection by RNA testing are the essential first steps along the treatment cascade [27]. The aim of this investigation was to clarify the contributing factors for engaging PWIDs in HCV testing.

## Methods

The present study was conducted based on the instructions in Protocols of Systematic Reviews and Meta-Analyses (PRISMA) [28, 29].

### Search strategy and study selection

**Fig. 1:**
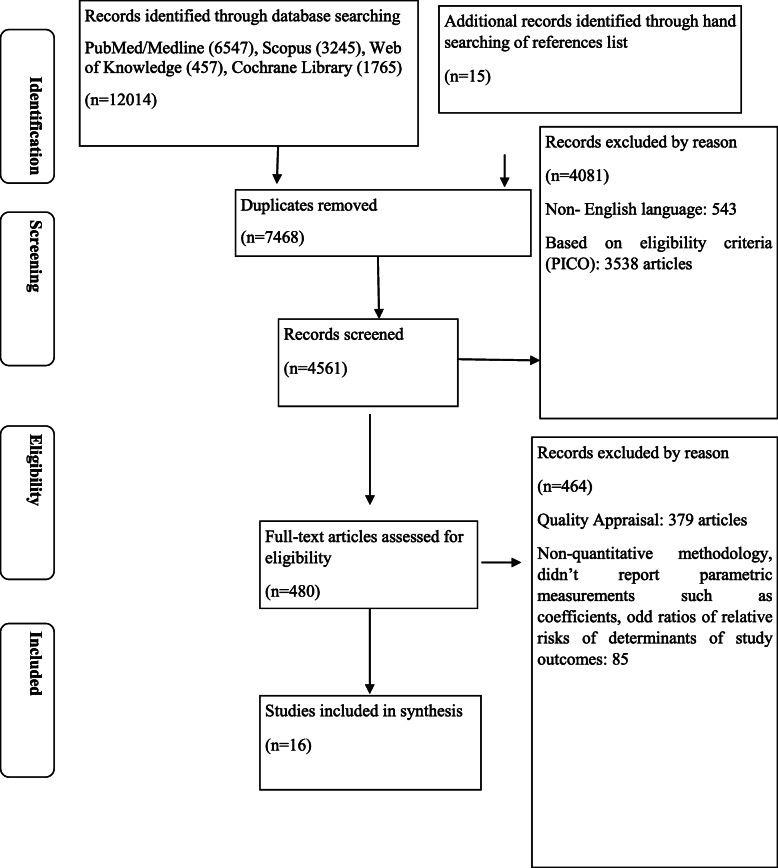
PRISMA flow diagram

The process of our study selection is presented in Fig. 1.

We selected 8348 papers from across 4 databases, and included additional manual searches of the reference lists for papers published before May 1, 2020. To identify and select studies two independent researchers **(A.B. and B.A.)** reviewed the electronic databases of PubMed, Scopus, Web of Science, and Cochrane independently (Table 1).

**Table 1:** search strategy

	PubMed
#18	Search ((((((((((((((((get HCV testing[Title/Abstract]) OR HCV counselling[Title/Abstract]) OR HCV testing[Title/Abstract]) OR rapid HCV testing[Title/Abstract]) OR HCV test[Title/Abstract]) OR HCV Testing Uptake[Title/Abstract]) OR HCV medical care[Title/Abstract]) OR HCV Diagnosis[Title/Abstract]) OR HCV status[Title/Abstract]) OR awareness of HCV status[Title/Abstract]) OR HCV Testing Behavior[Title/Abstract]) AND Persons Who Inject Drugs[Title/Abstract]) OR People Who Inject Drugs[Title/Abstract]) OR Inject Drug users[Title/Abstract]) OR PWID[Title/Abstract]) OR PWIDs[Title/Abstract]) OR Drug Users[MeSH Terms]
#17	Search Drug Users[MeSH Terms]
#16	Search PWIDs[Title/Abstract]
#15	Search PWID[Title/Abstract]
#14	Search Inject Drug users[Title/Abstract]
#13	Search People Who Inject Drugs[Title/Abstract]
#12	Search Persons Who Inject Drugs[Title/Abstract]
#11	Search HCV Testing Behavior[Title/Abstract]
#10	Search awareness of HCV status[Title/Abstract]
#9	Search HCV status[Title/Abstract]
#8	Search HCV Diagnosis[Title/Abstract]
#7	Search HCV medical care[Title/Abstract]
#6	Search HCV Testing Uptake[Title/Abstract]
#5	Search HCV test[Title/Abstract]
#4	Search rapid HCV testing[Title/Abstract]
#3	Search HCV testing[Title/Abstract]
#2	Search HCV counselling[Title/Abstract]
#1	Search get HCV testing[Title/Abstract]
	Scopus
	(TITLE-ABS-KEY (get AND HCV AND testing) OR TITLE-ABS-KEY (HCV AND counselling) OR TITLE-ABS-KEY (HCV AND testing) OR TITLE-ABS-KEY (rapid AND HCV AND testing) OR TITLE-ABS-KEY (HCV AND test) OR TITLE-ABS-KEY (HCV AND testing AND uptake) OR TITLE-ABS-KEY (HCV AND medical AND care) OR TITLE-ABS-KEY (HCV AND diagnosis) OR TITLE-ABS-KEY (HCV AND status) OR TITLE-ABS-KEY (awareness AND of AND HCV AND status) OR TITLE-ABS-KEY (HCV AND testing AND behavior) AND TITLE-ABS-KEY (persons AND who AND inject AND drugs) OR TITLE-ABS-KEY (people AND who AND inject AND drugs) OR TITLE-ABS-KEY (inject AND drug AND users) OR TITLE-ABS-KEY (drug AND users) OR TITLE-ABS-KEY (pwids) OR TITLE-ABS-KEY (pwid))
	web of knowledge
#1	TS = (get HCV testing OR HCV counselling OR HCV testing OR rapid HCV testing OR HCV test OR HCV Testing Uptake OR HCV medical care OR HCV Diagnosis OR HCV status awareness of HCV status OR HCV Testing Behavior)
#2	TS = (Persons Who Inject Drugs OR People Who Inject Drugs OR Inject Drug users OR PWID OR PWIDs OR Drug Users)
#3	#2 AND #1
	Cochrane
#1	(get HCV testing):ti,ab,kw (Word variations have been searched)
#2	(HCV counselling):ti,ab,kw (Word variations have been searched)
#3	(HCV testing):ti,ab,kw (Word variations have been searched)
#4	(rapid HCV testing):ti,ab,kw (Word variations have been searched)
#5	(HCV test):ti,ab,kw (Word variations have been searched)
#6	(HCV Testing Uptake):ti,ab,kw (Word variations have been searched)
#7	(HCV medical care):ti,ab,kw (Word variations have been searched)
#8	(HCV Diagnosis):ti,ab,kw (Word variations have been searched)
#9	(HCV status):ti,ab,kw (Word variations have been searched)
#10	(awareness of HCV status):ti,ab,kw (Word variations have been searched)
#11	(HCV Testing Behavior):ti,ab,kw (Word variations have been searched)
#12	(#1 OR #2 OR #3 OR #4 OR #5 OR #6 OR #7 OR #8 OR #9 OR #10 OR #11)
#13	(Persons Who Inject Drugs):ti,ab,kw (Word variations have been searched)
#14	(People Who Inject Drugs):ti,ab,kw (Word variations have been searched)
#15	(Inject Drug users):ti,ab,kw (Word variations have been searched)
#16	(PWID):ti,ab,kw (Word variations have been searched)
#17	(PWIDs):ti,ab,kw (Word variations have been searched)
#18	MeSH descriptor: [Drug Users] explode all trees
#19	(#13 OR #14 OR #15 OR #16 OR #17 OR #18)
#20	(#12 AND #19)

We included English language papers in the study, and also, we considered limitations such as time and geographic items. We reviewed papers twice based on the abstract and on the relevancy to the subject.

***Inclusion criteria based on PICOS:***

- **Population**: Individuals who report injecting drugs
- **Intervention**: HCV testing in the past year
- **The Comparison Group:** PWIDs who did not conduct HCV testing
- **Outcomes:** HCV testing among PWIDs.
- **Study design:** We included cross-sectional, cohort, and case-control studies.

We excluded qualitative studies, secondary studies, systematic reviews and, meta-analysis studies, and also, we excluded non-English language papers from our study.

### Data extraction and study quality assessment

Two independent researchers (**AB and BA**) reviewed and assessed the papers applying a standardized data collection form. Any contradictions between the two researchers were eliminated by discussing with two other members of the research team (**EA and YF**). We used Microsoft Excel software for data extraction and management. Two independent reviewers (author **BA and AB**) chose the references in a two-phased monitoring process. First, we eliminated duplicated titles/abstracts (89% agreement) according to the criteria one through three mentioned below. Second, titles/abstracts that met these initial criteria were selected for full-text review based on the inclusion criteria (96% agreement).

The authors gathered data from selected papers, including the surname of the first author, publication date, participant demographic data (age, sex, and level of education) and other characteristics like previous HCV testing, past treatment attempts, duration of injecting drug use and unprotected sex. The Newcastle-Ottawa Scale (NOS) [30] proposed by the Cochrane Collaboration [31] was used to evaluate the quality of the reviewed papers (Table 2).

**Table 2:** main characteristic of included studies

references	Country	Year	Sample Size	Study Design	Quality Assessment Criteria	Positive cases	OR Age > 30	Low CI Age > 30	High CI Age > 30	OR Female	Low CI Female	High CI Female	OR High school	Low CI High school	High CI High school	OR Unprotected sex	Low CI Unprotected sex	High CI Unprotected sex	OR Duration of injecting drug use	Low CI Duration of injecting drug use	High CI Duration of injecting drug use	OR Past treatment attempt	Low CI Past treatment attempt	High CI Past treatment attempt	OR Previous testing	Low CI Previous testing	High CI Previous testing
[32]	USA	2012	533	Cross-section	Good	469							1.9	1.4	2.5												
[33]	India	2015–2016	1241	Cross-section	Very Good	298							3.5	1.2	10.4	0.5	0.3	0.9	2.1	1	4.2						
[34]	Greece	2013–2015	2747	Cross-section	Very Good	2294	2.6	1.6	4.3										3	2	4.5	3.6	2.6	4.8			
[35]	Australia	2016–2018	565	Cohort	Very Good	367				1.92	1.11	3.3													2.15	0.97	4.8
[36]	Australia	2007	888	Cross-section	Good	826							0.36	0.18	0.81							2.78	1.48	5.2			
[37]	Thailand	2011	427	Cross-section	Very Good	141							2.2	1.35	3.64	0.62	0.32	0.97				3.47	1.85	6.95			
[38]	Ukraine	2014–2015	1613	Cross-section	Satisfactory	348				1.61	1.08	2.41							0.98	0.96	1	2.24	1.32	3.81			
[39]	Australia	215	1173	Cross-section	Good	633																2.15	1.149	3.87	2	1.31	3.06
[40]	Germany	2020	2059	Cross-section	Satisfactory	1503	2.2	1.2	4.2																		
[41]	India	2013–2017	11,993	Cross-section	Good																	1.72	1.22	2.43			
[42]	USA	2012–2013	129	Cross-section	Good	107	4.2	1.7	7.5																		
[43]	Spain	2011	240	Cross-section	Very Good	179																					
[42]	USA	2012–2013	129	Cross-section	Very Good	107																					
[44]	USA	2015	14,253	Cross-section	Good	157																					
[45]	Canada	2012–2013	663	Cross-section	Satisfactory	564																					
[46]	USA	2003–2004	217	Cross-section	Good	100																					
[47]	UK	2012–2015	211	Cross-section	Very good	135																					
[41]	India	2013–2017	11,993	Cross-section	Good	469																					

*OR* Odds Ratio

*CI* Confidence Interval

To evaluate the quality of papers included in the meta-analysis, we applied a modified version of NOS. This was used to evaluate statistical quality, sample representativeness, sample size, and comparability between people were the domains used for the NOS when evaluating the quality of individual studies. We applied agreement beyond chance (unweighted kappa) for evaluating agreement between the two authors (**BA and AB**) during the quality assessment. The levels of poor, slight, fair, moderate, substantial, and almost perfect levels of agreement were showed by the values 0, 01–0.02, 0.021–0.04, 0.041–0.06, 0.061–0.08, and 0.081–1.00, respectively [48].

### Data synthesis and statistical analysis

The present meta-analysis was conducted by generating pooled odds ratios (OR) and the 95% confidence intervals on recognizing factors associated with HCV testing among PWID. We computed the OR applying a 2*2 table, and we considered OR < 1 as a positive association between HCV testing and the target variable. An OR >1 (as the statistical threshold for assessing the relationship between outcome variables and expositive variables) suggests a protective relationship between variables and vice versa. To evaluate the lack of correlation between studies, we applied Q test with a *P* value < 0.05 and I^2^ statistics with a cutoff of ≥50%. We assessed 95% confidence intervals for I2 where we considered negative values as zero. We applied the random-effects model to calculate pooled estimation, considering the different sampling methods of the studies. To identify any publication bias, we used Begg’s and Egger’s publication bias approach both in graphical and statistical manners [49, 50]. *P*-values of less than 0.05 were considered significant. We demonstrated the association between social and demographical determinants by an OR and 95% CI, and showed the results in forest plots. For data analysis, we conducted the meta and metabias commands in STATA version 13.0 (STATA Corporation, College Station, TX.

## Results

### Study characteristics

After careful evaluations of the extracted citations, 16 studies were included [32–47]. Selected studies were from four WHO regions (five from Region of the Americas [*n* = 15,795 participants], five from the European Region [*n* = 6870 participants], three from South-East Asia [*n* = 13,661 participants] and three from the Western Pacific region [*n* = 2626 participants]. The USA had the highest number of reports (4 studies, 15,132 participants). Considering country income level, 12 studies (*n* = 23,678) were conducted within high-income countries, 1 study (*n* = 427) within upper-middle-income countries and three studies were (*n* = 14,847) from a lower middle-income country.

### Results of the meta-analysis

In Table 2, the key characteristics of the included studies for factors associated with HCV testing uptake among PWIDs are presented. The impact can be inferred through plots 2–9.

**Fig. 2:**
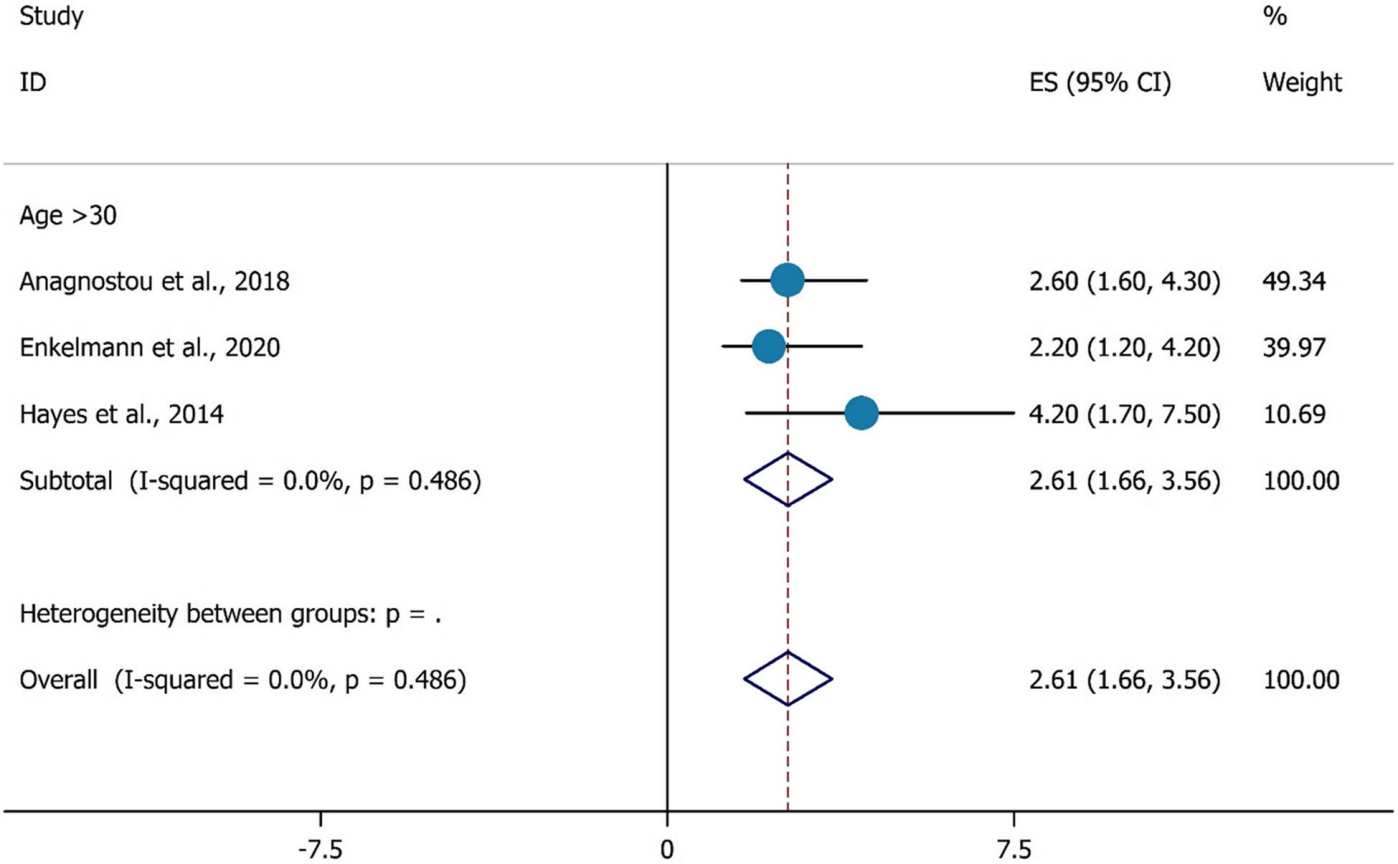
the association between age > 30 years with HCV testing uptake among PWID

Figure 2 indicates the association between being age > 30 years and HCV testing among PWIDs. Our findings show PWIDs aged > 30 years were 2.61 times more likely to report having had an HCV test during last year (OR = 2.61, 95%CI = 1.66–3.56).

**Fig. 3:**
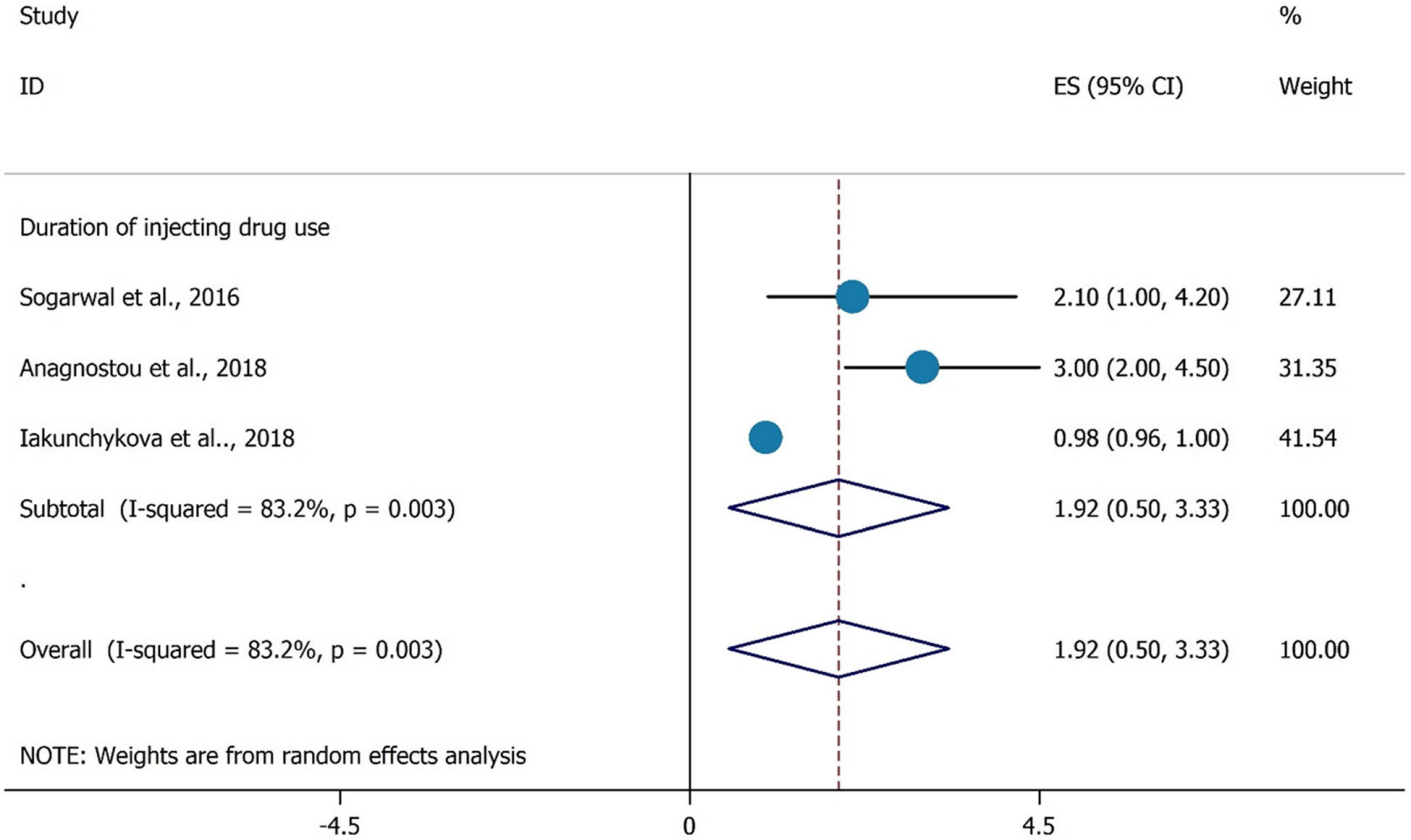
the association between duration of injecting drug use with HCV testing uptake among PWID

**Fig. 4:**
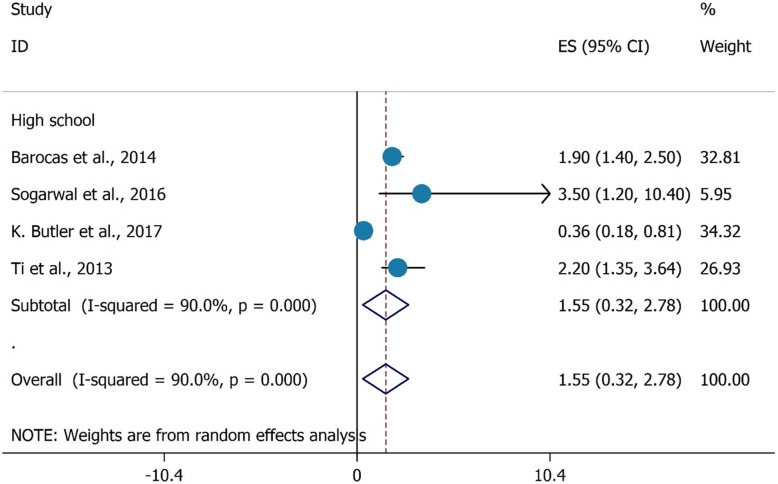
the association between had high school education with HCV testing uptake among PWID

As illustrated in Figs. 3, and 4, there were no significant associations found with HCV testing among PWIDs and their duration of injecting (OR = 1.92, 95%CI = 0.50–3.33), and high school education (OR = 1.55, 95%CI = 0.32–2.78).

**Fig. 5:**
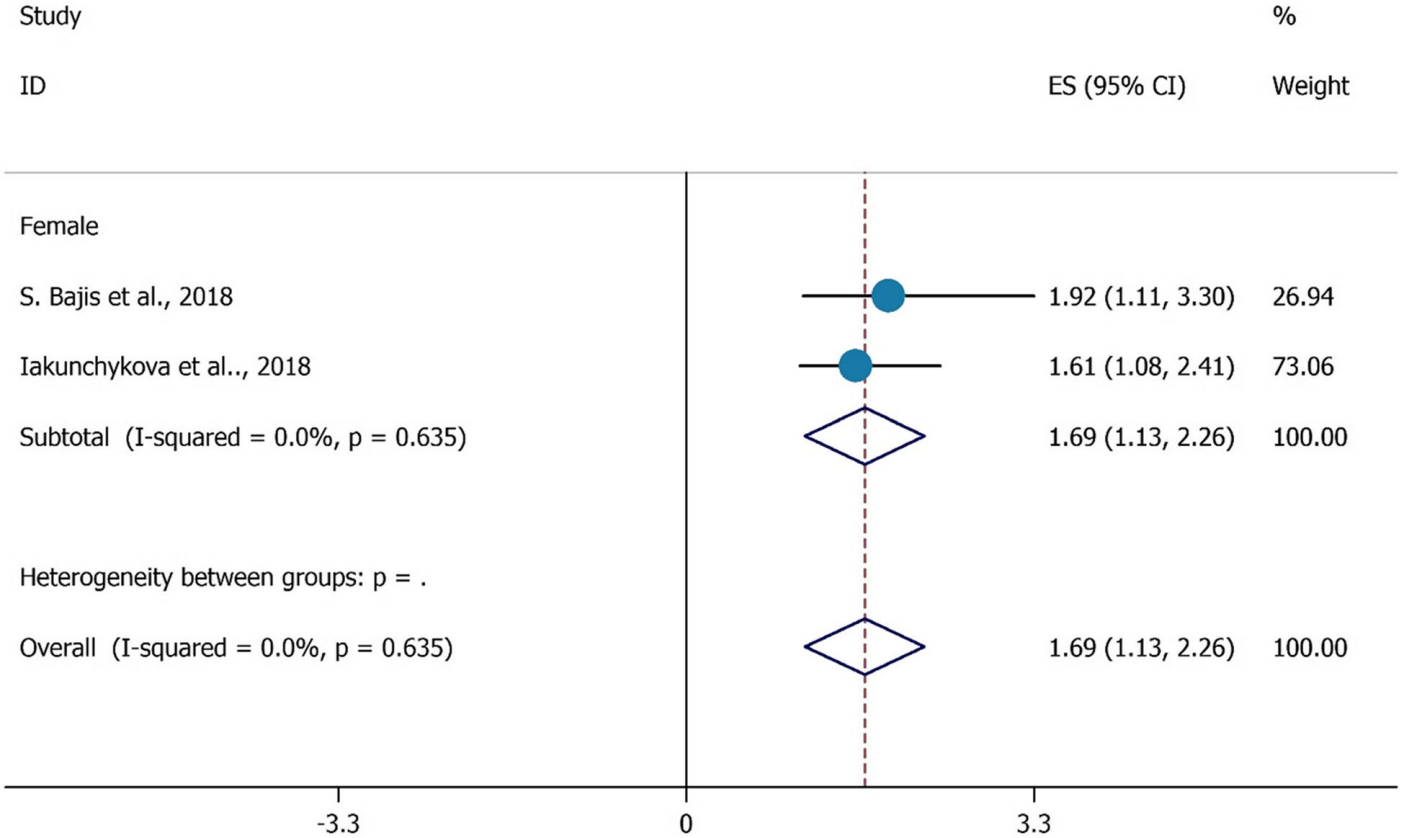
the association between being female with HCV testing uptake among PWID

Figure 5 shows the positive impact of being female and HCV testing among PWIDs, and the overall heterogeneity was about 0%. The pooled effect size has a respective positive impact and the lower boundary is about 1.13, and the higher is 2.26. The OR indicated a positive and effective role that being female has on HCV testing among PWIDs. Participants who were female were 1.69 times more likely to test for HCV in the last 12 months (OR = 1.69, 95%CI = 1.13, 2.26).

**Fig. 6:**
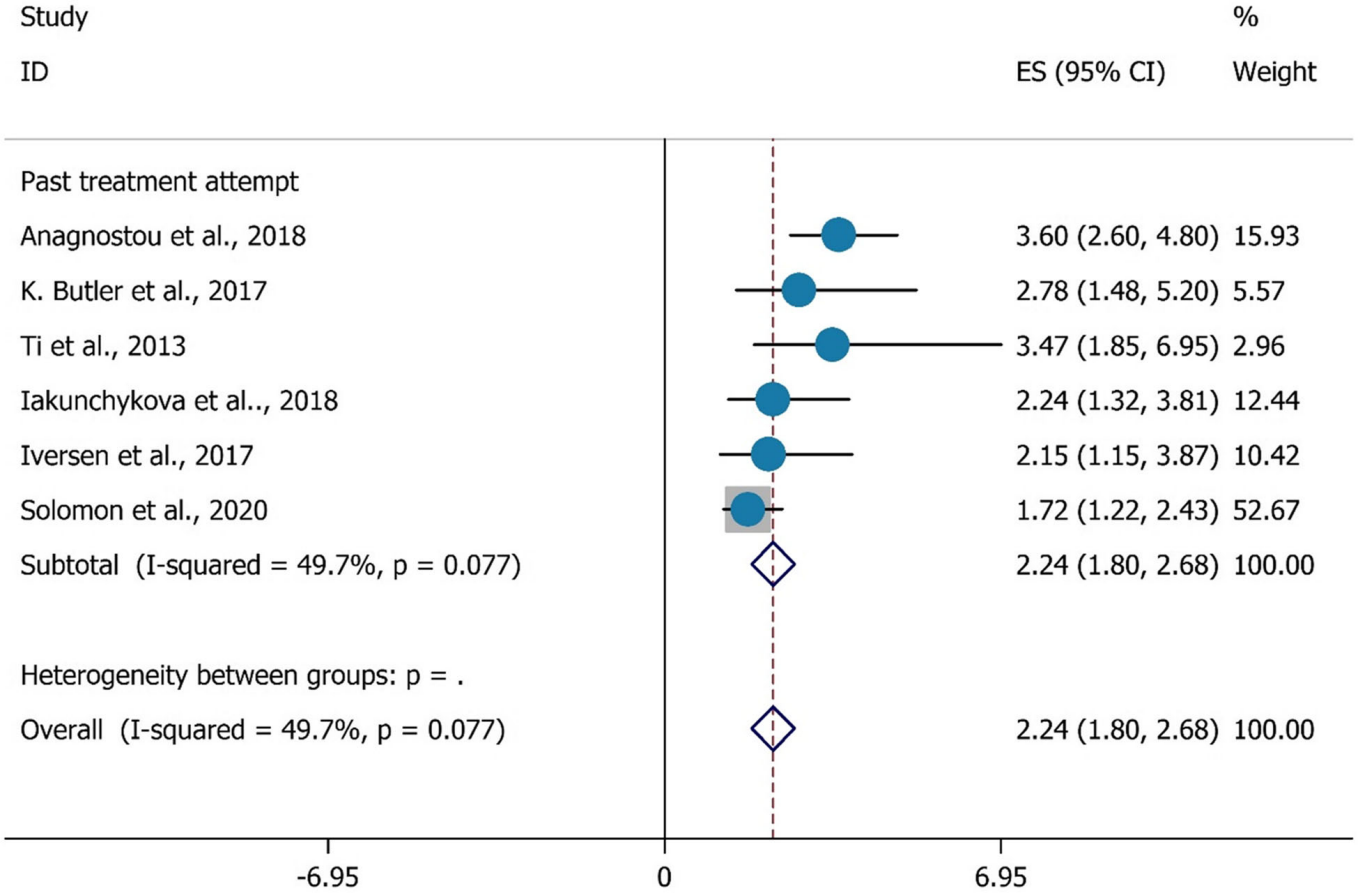
The association between past treatment attempt with HCV testing uptake among PWID

Additionally, Fig. 6 indicates a significant correlation between past treatment attempts and HCV testing among PWID. PWIDs in this situation were 2.24 times more likely to have had an HCV test in the previous 12 months than PWIDs who did not report any past treatment attempts (OR = 2.24, 95%CI = 1.80–2.68).

**Fig. 7:**
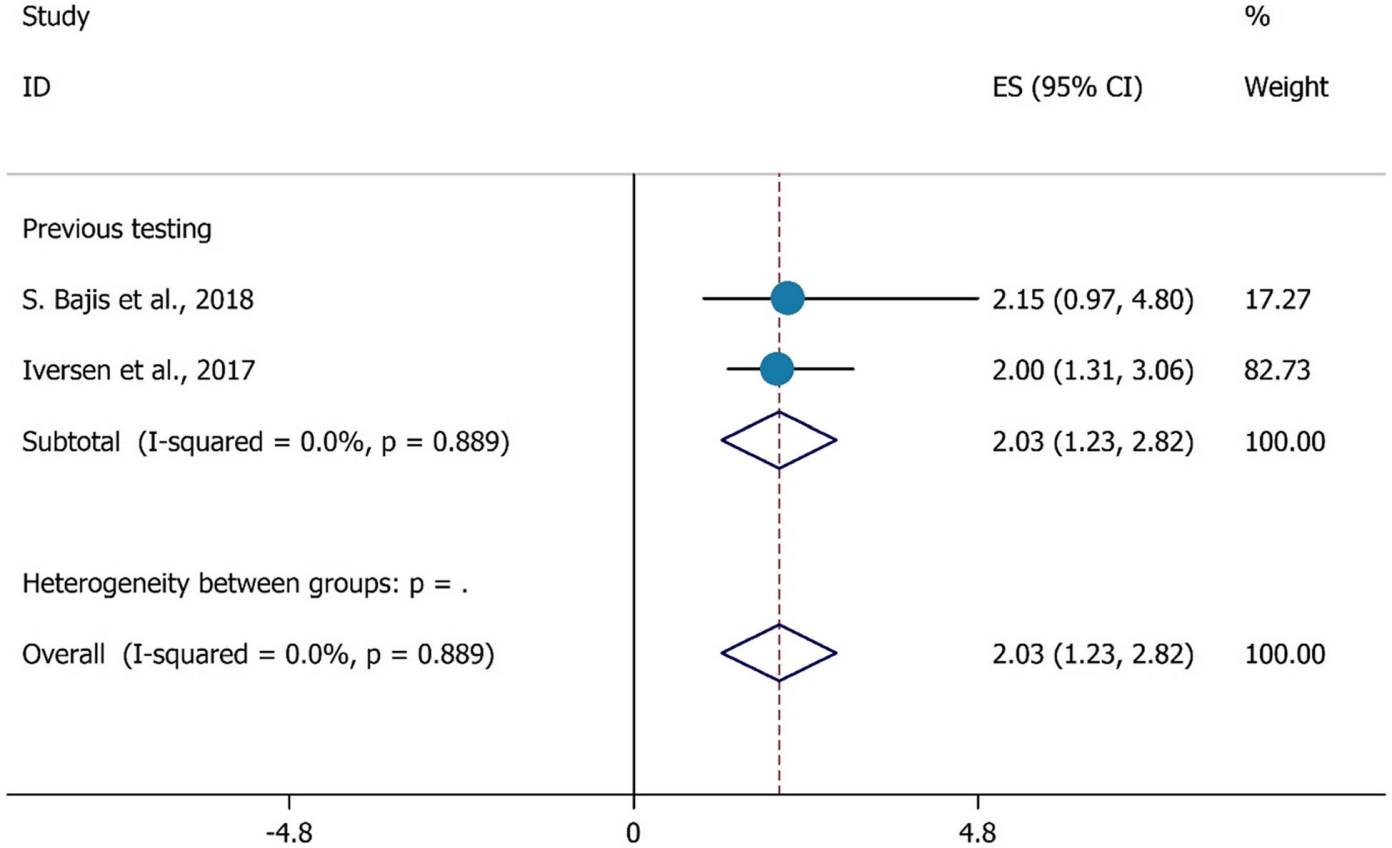
The association between previous HCV testing with HCV testing uptake among PWID

Another finding from our data was previous testing had a positive association with HCV testing. Those PWIDs who had a previous test were 2.03 times more likely to have completed a recent HCV test (last 12 months) (OR = 2.03, 95%CI = 1.23–2.82) (Fig. 7).

**Fig. 8:**
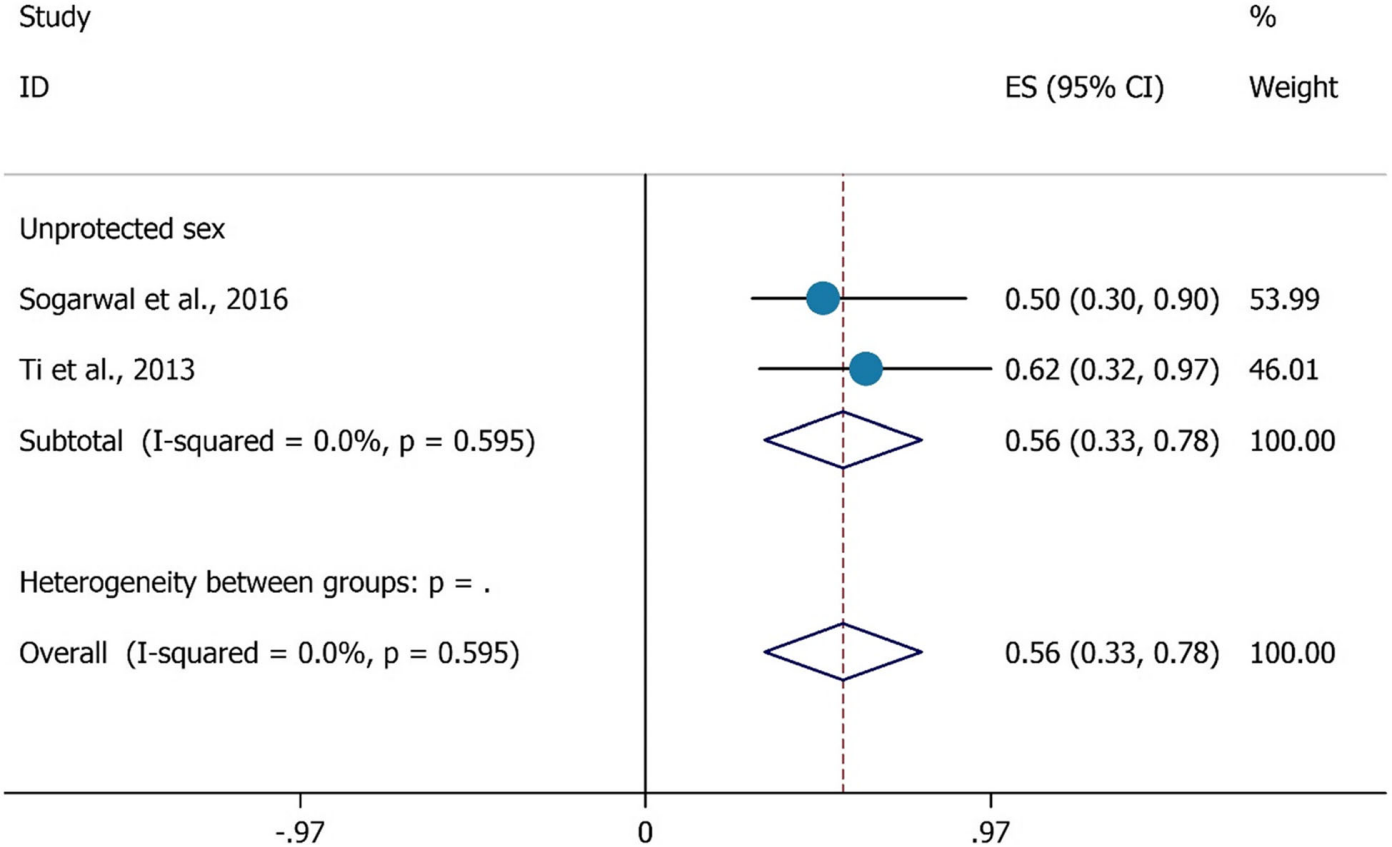
The association between had unprotected sex with HCV testing uptake among PWID

Finding of present study was that unprotected sex had a negative association with HCV testing. Those PWIDs who had unprotected sex were 0.56 times less likely to have completed HCV testing during last year (OR = 0.56, 95%CI = 0.33–0.78) (Fig. 8).

**Fig. 9:**
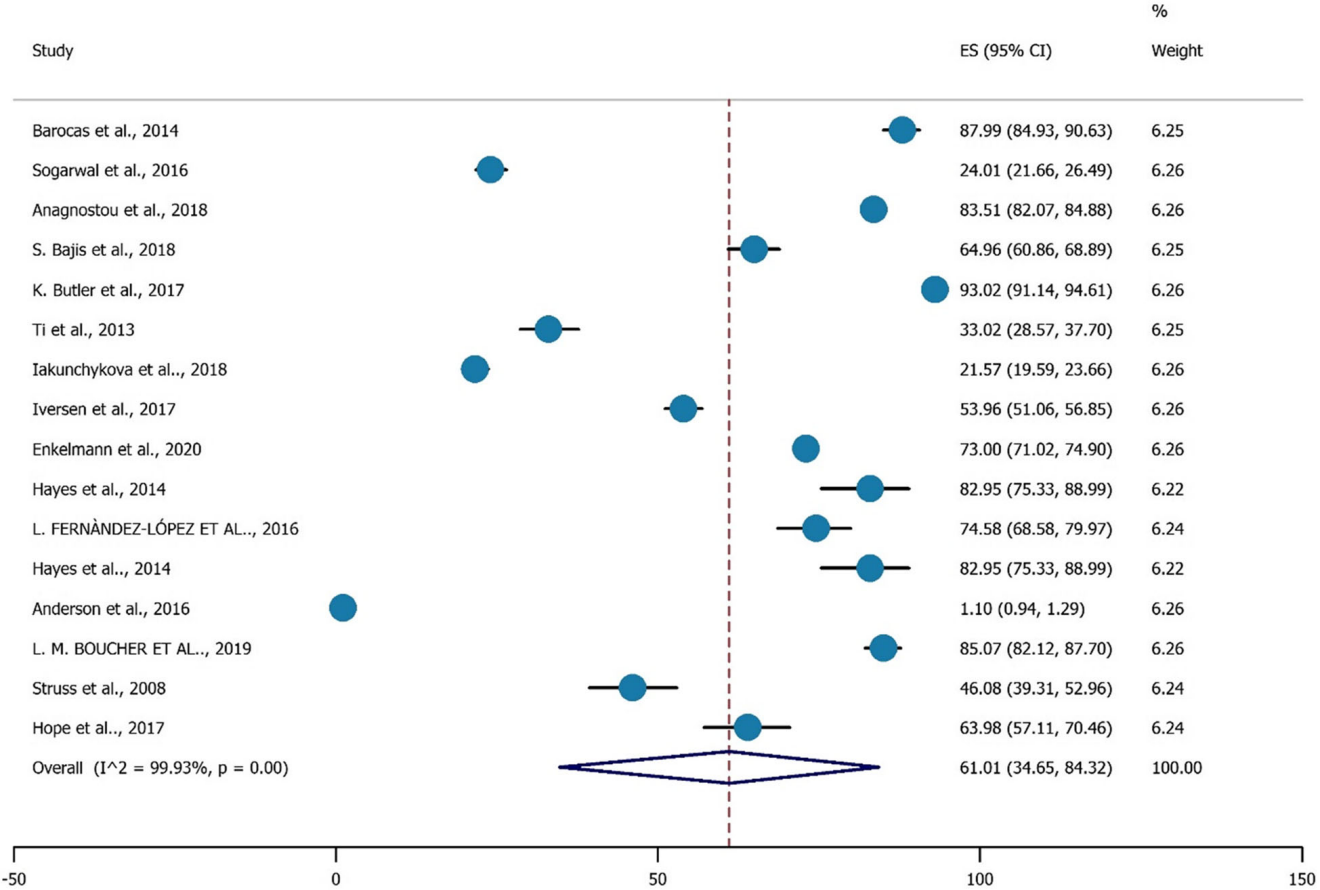
The prevalence of HCV testing uptake among PWID

Figure 9 shows a significant pooled prevalence of 61.01% (95% CI, %34.65-%84.32) for the prevalence of HCV testing among PWIDs.

**Fig. 10:**
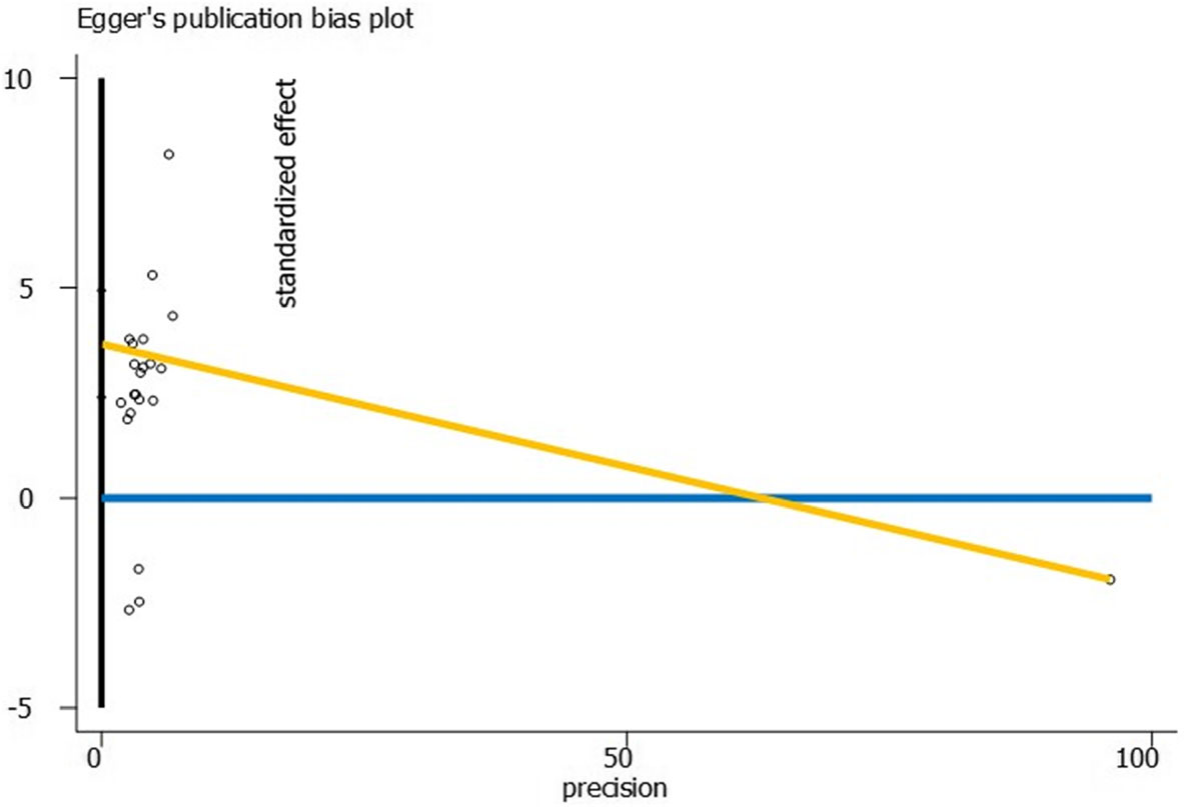
The Eggers plot for detect publication bias among studies

To identify the probable publication bias, the Egger’s test and the graph were performed. Considering the symmetry assumption, there was no significant publication bias in the reviewed studies selected for inclusion. As regards the funnel plot, the distribution of the articles was not oriented and for most of them, it was identical confirming no publication biases observed in our study. The publication bias test indicates considerable bias based on Eggers test (coefficient = 3.66, *P* value < 0.001) (Fig. 10).

## Discussion

This meta-analysis study aimed to explore factors associated with recent HCV testing among PWIDs. The prevalence of recent HCV testing uptake was high. Past 12 months testing for HCV was associated with being aged > 30 years, being female, having a past treatment attempt and reporting previous HCV testing. HCV testing was reported by 61.01% of the study participants which could indicate enhanced access to harm reduction interventions. Many drug treatment services require initial HCV screening for PWIDs and such services are in the perfect position to conduct long-term follow-up and monitoring of HCV testing in this population.

Moreover, a strong predictor for a recent HCV test was being over the age of30 years. Given the correlation between a longer duration of drug injecting and the increased odds of high-risk injecting. This may reflect an association between increased HCV test-taking and longer injecting history. However, studies also show that HCV infection can also occur in the early stages of people’s injecting careers [51]. While the risks are high in this group evidence suggests they ae also more likely to be conscious of the advantages in HCV testing [52, 53]. In addition, while recent HCV testing and female gender were independently correlated the risks are more challenging to manage. Sex work and injection-assistance [54] have been shown to increase vulnerability to HCV infection [38].

Addressing injecting couple’s relationships in terms of developing and conducting HCV testing and considering gender-specific needs are necessary if increased HCV testing is to be achieved [55]. Our study suggests that accessing drug treatment by PWIDs is a determining factor in recent HCV test-taking. In line with this result, other studies have highlighted the advantages of drug treatment in facilitating access to healthcare services for PWIDs [56, 57] especially HIV and HCV treatment programs [58]. However, when excluding treatment from the variable definition, drug treatment and recent HCV testing were not associated and this may be a result of inadequate staff training on the risks and transmission methods of HCV.

There is evidence that providing education programs for drug treatment staff aimed at improving the HCV awareness i is beneficial in promoting HCV services among PWID [59]. HIV service integration highlights the need for developing strategies due to increased demands [9, 60]. Such plans could help to improve the HCV-related knowledge of the general population, which in turn results in eliminating targets. Network-based recruitment and the use of PWID peer facilitators might enhance the detection rates of HCV cases [61–63]. Accordingly, network referral strategies could be combined to increase HCV testing and treatment at the community level.

Furthermore, there was an independent association between confirmatory PCR testing and undergoing HCV testing within the past 12 months. Enhanced awareness of the need for confirmatory testing in healthcare providers and PWIDs is essential if WHO elimination goals are to be achieved. Approximately 33% of PWIDs with histories of active HCV infection had received HCV professional surveys (e.g., liver biopsy or FibroScan1) before being introduced to interferon-free regimens. However, there is a significant gap between healthcare engagement and the receipt of HCV treatment in the era of interferon-based therapy. It is reported that 1–2% of PWIDs received treatment per year. Additionally, a cumulative therapy of 10% at the end of 2011 are extensively in line with these data [39]. Consistent with prior research [33, 39] we found that having unprotected sex was negatively associated with having been tested for HCV. There is a data discrepancy on HIV-related counseling programs and undertaking the relevant test in terms of high-risk sexual behaviors. A tremendous body of literature suggested a major decline in claiming multiple partnerships and unprotected sexual relationships. On the other hand, some investigators [64–66], documented either adverse or poor effects of test-taking on behavioral modifications [67, 68]. Sex-related high-risk behaviors, such as the lack of using sexual protection tools and fisting, which in turn, could be mucosal traumatic and result in bleeding. These factors have also been suggested to be related to contracting HCV risk [69–71]. It is still debatable whether bleeding plays an essential role in the transmission of HCV. Some investigations have detected HCV in seminal and rectal fluids in males who were infected with HIV and supported that such fluids could interfere with the transmission of HCV [72, 73].

### Limitations and strengths of the study

The study limitations include first, relying on HCV self-reports (study samples might have failed to completely percept the HCV diagnosis approach, i.e., comprising 2 test forms, the reported data may be biased by overestimating). Although not commonly implemented such limitations could be eliminated by achieving confirmation of prior HCV testing. Second, most of the included studies were cross-sectional meaning causal and temporal relationships between risk behavior and HCV testing are not possible. However, this meta-analyses may enhance the statistical inference of analyses and incresase the reliability of the evidence. Third, few studies investigated the association between risk behavior and HCV testing, emphasizing this gap in the literature. Also, since we did not interfere with the setting of independent and dependent variables, we had to report only the data that were published in the articles. A.

The strengths of our study include the number of high-quality studies reviewed, the large representative sample and the multivariate analysis which regulaed for potential confounders. These factors provided greater statistical power and strengthened the results of the reviewed studies and enhanced the chance of recognizing a true effect of exposure [74]. Longitudinally design research aiming at chronic HCV infection detection as well as HCV treatment enrollment, are warranted in PWIDs.

## Conclusion

The findings of the present study illustrate the important factors that may be effective to increase HCV testing rates among PWID, which may improve prevention and reduce transmission. This study concludes that better understand the social determinants of injecting risk provides an area for exploring effective interventions to improve HCV testing practices and individual risk reduction. As such the integration of HCV testing and treatment within exisiting low threshold harm reduction programs could be a possible solution to address the HCV burden among PWID and ultimately helping to reach the WHO goal of global elimination.

## Data Availability

The datasets used and/or analyzed during the current study are available from the corresponding author on reasonable request.

## Abbreviations

CI: Confidence intervals
HCV: Hepatitis C virus
NSPs: Needle and syringe programmes
NOS: Newcastle-Ottawa Scale
OR: Odds ratio
PICO: Population, Intervention, Comparator, Outcomes
PRISMA: Protocols of Systematic Reviews and Meta-Analyses
PWIDs: People who inject drugs
WHO: World Health Organization
